# Active Magnetoelectric Motion Sensing: Examining Performance Metrics with an Experimental Setup

**DOI:** 10.3390/s21238000

**Published:** 2021-11-30

**Authors:** Johannes Hoffmann, Eric Elzenheimer, Christin Bald, Clint Hansen, Walter Maetzler, Gerhard Schmidt

**Affiliations:** 1 Institute of Electrical Engineering and Information Technology, Faculty of Engineering, Kiel University, 24143 Kiel, Germany; jph@tf.uni-kiel.de (J.H.); ee@tf.uni-kiel.de (E.E.); cbal@tf.uni-kiel.de (C.B.); 2 Department of Neurology, Kiel University, 24105 Kiel, Germany; c.hansen@neurologie.uni-kiel.de (C.H.); w.maetzler@neurologie.uni-kiel.de (W.M.)

**Keywords:** motion tracking, magnetoelectric sensors, artificial fields

## Abstract

Magnetoelectric (ME) sensors with a form factor of a few millimeters offer a comparatively low magnetic noise density of a few pT/$\sqrt{\mathrm{Hz}}$ in a narrow frequency band near the first bending mode. While a high resonance frequency (kHz range) and limited bandwidth present a challenge to biomagnetic measurements, they can potentially be exploited in indirect sensing of non-magnetic quantities, where artificial magnetic sources are applicable. In this paper, we present the novel concept of an active magnetic motion sensing system optimized for ME sensors. Based on the signal chain, we investigated and quantified key drivers of the signal-to-noise ratio (SNR), which is closely related to sensor noise and bandwidth. These considerations were demonstrated by corresponding measurements in a simplified one-dimensional motion setup. Accordingly, we introduced a customized filter structure that enables a flexible bandwidth selection as well as a frequency-based separation of multiple artificial sources. Both design goals target the prospective application of ME sensors in medical movement analysis, where a multitude of distributed sensors and sources might be applied.

## 1. Introduction

Magnetic sensing is well established in movement analysis [1,2] as most inertial measurement units (IMUs) contain 3D magnetometers to determine the unit’s orientation in the horizontal plane (compass). Common scientific applications include human movement analysis, where medical doctors use motion tracking systems in both diagnosis and therapy of neurodegenerative disorders with movement-related symptoms: sensors are commonly applied in a lab-based assessment to examine early motor markers of Parkinson’s disease [3]. Patient monitoring in the home setting is another emerging approach that relies heavily on wearable sensor technology [4,5].

Active magnetic motion tracking shows the potential to be a supplemental source of reference data [6]. In comparison to the passive magnetic method, it utilizes artificial magnetic fields of excitation coils in combination with tracking algorithms to obtain the relative position and orientation data between source and sensor [7]. Integrated commercial magnetometers based on Hall-effect sensors are well-suited for pure geomagnetic applications (up to 50 μT), as they offer sufficient noise performance (e.g., below 0.3 μT for BMX005, Bosch Sensortec [8]) at low cost and small integration size. For active magnetic motion tracking, sensor performance is of much greater concern, as actuators (excitation coils) consume significant amounts of power to generate magnetic fields, which is noticeable in the large required currents (e.g., 1.5 A [6]). A low power consumption is generally desirable as it improves runtime for battery-powered applications. In essence, the required power to reach a given signal-to-noise ratio (SNR) at a given distance (and orientation) can be significantly decreased by the selection of a performant sensor type in combination with optimized transmission and readout schemes.

In this contribution, we employ magnetoelectric (ME) delta-E-effect sensors [9] in direct detection of the first bending mode (approximately 7.7 kHz) with a magnetic noise density below 10 pT/$\sqrt{\mathrm{Hz}}$ in resonance. Assuming mono-frequent signals at a sensor-optimized frequency, the sensors perform in the same order of magnitude as fluxgate sensors (e.g., FL1-100, Stefan Mayer Instruments: 20 pT/$\sqrt{\mathrm{Hz}}$ at 1 Hz [10]) and slightly inferior to total field optically pumped magnetometers (e.g., QTFM, QuSpin: below 1 pT/$\sqrt{\mathrm{Hz}}$ [11]). However, OPMs require heating power (e.g., total field OPM, Twinleaf: 0.7 W [12]) for laser and cell temperature stabilization, which might be undesirable in body-worn setups. Better performing sensors like superconducting quantum interference devices (SQUIDs) are not considered here, as they bring the significant drawbacks of supercooling (SQUIDs) or magnetically shielded operation (zero field OPMs). Aside from sensor noise, requirements differ heavily between applications as the term “motion sensing” is used interchangeably in indoor tracking/navigation, localization, and motion tracking of humans. Relevant performance metrics include measurement volume, (stationary) spatial accuracy, and update rate/bandwidth.

Indoor tracking or navigation systems as in [13,14] offer a measurement volume of multiple cubic meters aided by IMUs. In contrast, specialized devices for the tracking of medical instruments (e.g., Polhemus Viper [15]) target a much smaller volume and achieve sub-millimeter accuracy for the stationary case. Depending on actuator and sensor, different frequency ranges can be targeted: direct current (dc) systems have to compensate for the geomagnetic field and other stray fields (hard iron) [16]. Alternating current (AC) systems [15] might operate in the very low frequency (VLF) band from 3 kHz to 30 kHz, which is also suitable for the delta-E sensor type. Such systems avoid the geomagnetic field and some DC stray fields but have to deal with eddy currents in nearby conductive materials [17]. Due to their propagation velocity at the speed of light and their wavelengths in the 10 km range, these VLF approaches do not suffer from multi-path propagation and the Doppler effect [7], which results in a straight forward channel model for the stationary case. However, this does not eliminate the impact of relative movement in the context of complex field geometries.

Motion tracking systems commonly (e.g., [13]) use stationary sources in combination with distributed sensors. As most systems use multiple (orthogonal) sources, some form of multiple-access technique has to be employed to divide the source signals at the sensor. The sources might therefore operate sequentially as in [6] (time division multiple access, TDMA), by applying spreading codes (code division multiple access, CDMA) [13] or in separate frequency bands (frequency division multiple access, FDMA), such as the magnetoelectric approach presented here. The operation of multiple interoperable transmitters and receivers potentially enables powerful tracking approaches, as each additional unit results in a multitude of new data points. For a number of *L* sources and *M* sensors, this results in L×M data points.

This article focuses on the sensing of magnetic fields where characteristics of excitation, field, sensor, and basic signal enhancement are considered. This does not include tracking and sensor fusion algorithms [14], which are typically the following processing stages and can contribute to a significantly improved overall performance.

This paper is structured as follows: Section 2 details the characterization of the components and their mathematical description in the system. Section 3 introduces the experimental setup. It also features the acquired signals for the single channel and the FDMA approach as well as noise measurements and derived performance metrics. Section 4 contains a conclusion and an outlook.

## 2. System Characterization and Simulation

**Figure 1:**
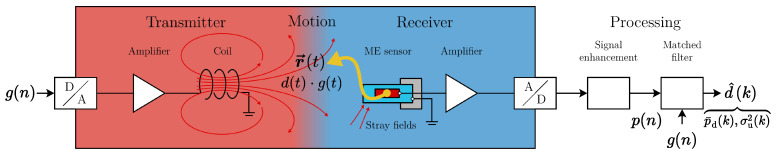
Magnetic motion sensing system overview.

The magnetoelectric motion sensing system (Figure 1) consists of three subsystems:

(1) The transmitter generates a magnetic AC field by feeding a periodic excitation signal g(n) (zero-mean, normalized power) through an amplifier to a coil.
(2) The receiver (1D sensor, fixed orientation) moves on a trajectory $\vec{r}\left(t\right)$ relative to the transmitter unit. Thus, it senses the excitation signal g(t) weighted with the magnetic flux density d(t) (desired signal) along the trajectory. This behavior resembles an amplitude modulation.
(3) The signal processing system’s task is to estimate an unbiased $\hat{d}\left(k\right)$ in a frame-based fashion with the subsampled time index *k*. Therefore, prior knowledge on the sensor system (signal enhancement) and the excitation sequence (matched filter) is applied.

The mathematical description of our approach is based on a distinction between the periodic excitation signal g(n) and the discrete desired signal d(n), which corresponds to a DC magnetic field with time variance induced by motion. The central component of the enhanced signal p(n) is the modulation term (with minor deviations between discrete and continuous signals omitted). Undesired signal components like stray fields and sensor intrinsic noise are represented in the aggregated noise term $u_{p}\left(n\right)$:

(1) $$p\left(n\right)=d\left(n\right)\;g\left(n\right)+u_{p}\left(n\right).$$

The matched filter separates the desired signal d(n) from the excitation signal g(n). Therefore, the output estimation is comprised of only the desired signal power ${\bar{p}}_{d}\left(k\right)$ and the noise power $\sigma_{u}^{2}\left(k\right)$ (zero-mean). Thus, the resulting signal-to-noise ratio (SNR) is independent of the excitation signal waveform:

(2) $$SNR(k)\;=\frac{{\bar{p}}_{d}\left(k\right)}{\sigma_{u}^{2}\left(k\right)}.$$

We introduce a position-dependent SNR for spatial performance considerations that only depends on the sensor position $\vec{r}$ at a given moment in time, without prior knowledge of the trajectory. Its definition requires multiple assumptions:

(1) The period of the highest frequency component in the desired signal $T_{d}^{\max}$ is much longer than the temporal length of the matched filter $T_{\mathrm{mf}}$. This leads to a desired signal power $d^{2}\left(\vec{r}\right)$ which is approximately constant within a frame.
(2) The noise power is both space- and time-invariant within the measurement area and duration.

(3) $$SNR(\vec{r})\;=\frac{d^{2}\left(\vec{r}\right)}{\sigma_{u}^{2}}$$

The following subsections focus on the contributions of the source and the sensor system regarding this metric in the system’s operating frequency range near the sensor’s resonance frequency $f_{0}$ of approximately 7.7 kHz.

### 2.1. Source Selection and Limitations

As a setup with multiple homogeneous or linear gradient fields is difficult to employ in a scenario with distributed moving sources, we used small cylindrical air coils. Manufacturing was done by spooling approximately 80 windings ($N_{w}$) of 0.5 mm enameled copper wire on a 3D-printed plastic body. The medium radius *R* was approximately 6.7 mm.

**Figure 2:**
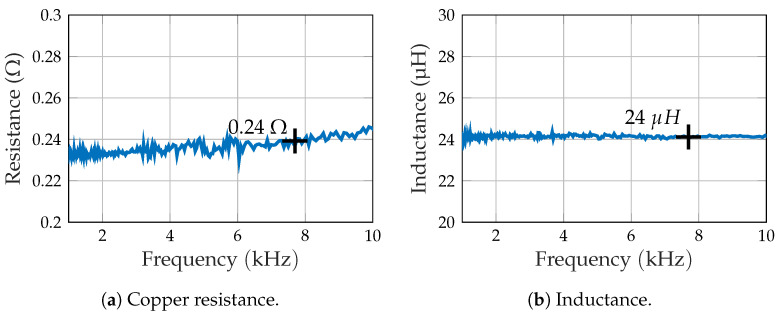
Impedance of the primary excitation coil.

According to Ampere’s circuital law [17], the magnetic flux density of a conductor loop is driven by the current $I_{\mathrm{rms}}$. For a specified input voltage (voltage source) $I_{\mathrm{rms}}$ is limited by the electrical impedance of the coil, whose equivalent circuit is assumed here as a copper resistance in series with an inductance. Both parameters of the primarily used coil in the relevant frequency range were measured with a vector network analyzer (Bode 100, Omnicron Labs [18]). The resistance (Figure 2a) is close to 0.24 Ω at $f_{0}$, while the inductance (Figure 2b) is almost constant with 24 μH. These comparatively low values have to be considered with regard to accurate current measurements.

The resulting magnetic flux density $\vec{b}$ of the coil is also affected by its physical properties. As a universal approach, the law of Biot–Savart [17] can be applied for the field computation of an arbitrary ensemble of line conductors. While there are analytic solutions for a simple cylindrical helix coil [19], a more complex coil might require a computationally intensive discrete model.

The magnetic dipole equation [20] offers a lightweight alternative, assuming the displacement *r* is much greater than the medium radius of the coil *R*. The actual distance requirement is dependent on the coil and the acceptable imitation error and might lay between 4 and 10 *R* [21]. The magnetic flux density $\vec{b}\left(\vec{r}\right)$ at a specified position $\vec{r}$ depends on the vacuum permeability $\mu_{0}$, and the coil’s magnetic dipole moment $\vec{m}$ with the number of windings *N*. Each vector ($\vec{r}$, $\vec{m}$) is comprised of the vector norm (*r*, *m*) and the corresponding unit vector $\left({\vec{e}}_{r},{\vec{e}}_{m}\right)$. This equation describes a time-invariant (DC) magnetic field, which yields an AC field, when modulated with the excitation signal g(n):

(4) $$\vec{b}\left(\vec{r}\right)\;=\frac{\mu_{0}}{4\pi r^{2}}\;\;\cdot \;\frac{3\;\vec{r}\left(\vec{m}\circ \vec{r}\right)-\vec{m}\;r^{2}}{r^{3}}\;\mathrm{with}\;\vec{m}=N_{w}\;I_{\mathrm{rms}}\;R^{2}\;\pi \;{\vec{e}}_{m}.$$

The magnetic flux density at the sensor’s position is comprised of constant, distance-dependent, and directivity-related contributions. This results in a nominal flux density $b_{0}$ with a field constant $a_{0}$, a 1/$r^{3}$ decay (path loss), and a corresponding loss due to directivity $\vec{\Theta }\left({\vec{e}}_{r}\right)$.

(5) $$\vec{b}\left(\vec{r}\right)\;=\underset{b_{0}}{\underset{︸}{a_{0}\;I_{\mathrm{rms}}}}\;\;\cdot \;\;\frac{1}{r^{3}}\;\;\cdot \;\;\vec{\Theta }\left({\vec{e}}_{r}\right)$$

The nominal field $b_{0}$ determines the feasible magnetic flux density at a specified position. It is constrained by competing geometric and electric parameters, as only a limited number of windings $N_{w}$ at a radius *R* fit into a constrained space. A reduced wire cross section enables more windings, but increases the electrical impedance and vice versa. An increase in current is generally possible within the voltage range of the source, but is ultimately limited by thermal constraints.

### 2.2. Sensor Characterization and Idealization

This motion sensing approach uses exchange bias magnetoelectric delta-E cantilever sensors with a sensor element made of multiple layers of magnetostrictive FeCoSiB material and piezoelectric material on a silicon substrate [22]. A sensor element and a low-noise charge amplifier [23] together form the ME sensor system.

The ME cantilever sensor is a vector magnetometer [24]. Accordingly, it will pick up magnetic fields depending on the orientation of its sensitive axis ${\vec{e}}_{s}$. Fabrication might cause a tilt between the physical long axis and the sensitive axis. Based on our observations while rotating the sensor in a Helmholtz coil, the tilt is very small (low single digit degrees maximum) for this specific sensor. Thus, the magnetic flux density $d(\vec{r})$ that the sensor picks up due to modulation might be modeled as the dot product of the sensor orientation and the magnetic vector field:

(6) $$d\left(\vec{r}\right)={\vec{e}}_{s}\circ \vec{b}\left(\vec{r}\right).$$

The sensor characterization measurements were done in the optimized conditions of a magnetically shielded measurement setup [25]. Therefore, real-world measurements will show an inferior sensor performance. The selected frequency space is targeted at the first bending mode close to 7.7 kHz, which is specified by the mechanical properties of the cantilever. Delta-E effect readout schemes for much lower frequencies [9] are also possible for this type of sensor, but not used in this application.

**Figure 3:**
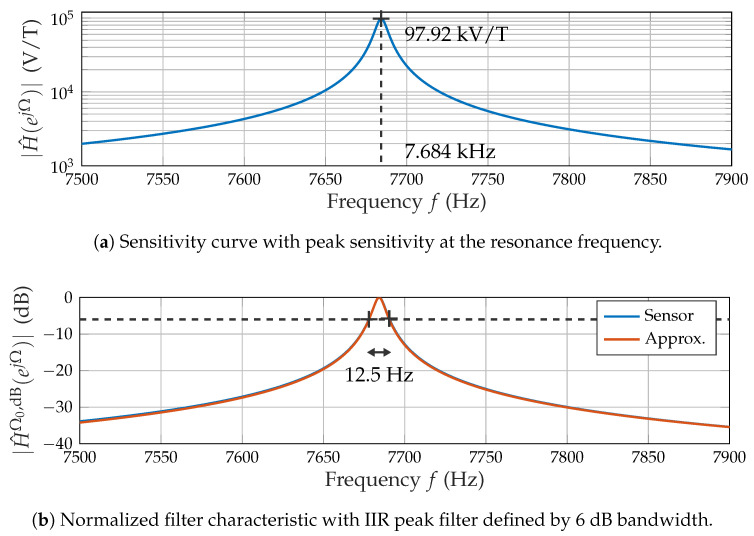
Frequency response of the ME sensor.

Based on the assumption of Linearity and time invariance (LTI), the measured frequency response $\hat{H}\left(e^{j\Omega }\right)$ (Figure 3a) quantifies the conversion of a magnetic field into a voltage. It is synonymous with the sensor’s sensitivity in this context.

The sensitivity curve (Figure 3a) results from multiple discrete measurement points interpolated using a spline method. The data were obtained using a magnetic sine sweep at a peak amplitude of 100 nT. The peak sensitivity (approximately 100 kV/T) is reached at the resonance frequency (approximately 7.684 kHz). The filter characteristic (Figure 3b) of the sensor is obtained from the logarithmic amplitude response normalized to the peak sensitivity ${\hat{H}}^{\Omega_{0},\mathrm{dB}}\left(e^{j\Omega }\right)$. The terminology for discrete-time signals with the normalized angular frequency Ω and the corresponding resonance frequency $\Omega_{0}$ leads to:

(7) $$|{\hat{H}}^{\Omega_{0},\mathrm{dB}}\left(e^{j\Omega }\right)|=20\;\log_{10}\left(\frac{|\hat{H}\left(e^{j\Omega }\right)|}{|\hat{H}\left(e^{j\Omega_{0}}\right)|}\right).$$

The resulting 6 dB bandwidth of 12.5 Hz and the resonance frequency were adopted to design a digital filter that approximates the filter characteristic in the region of interest. Based on a priori knowledge of the mechanical cantilever, an infinite impulse response (IIR) peak (resonator) filter [26] was chosen (Figure 3b).

**Figure 4:**
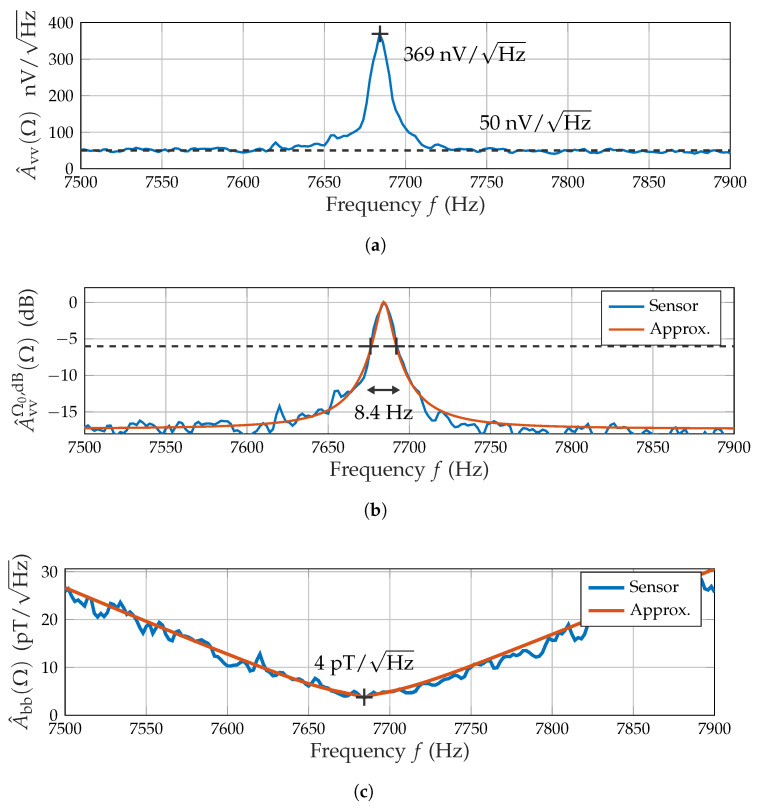
ME sensor noise characteristics. (**a**) Voltage noise spectral density with peak and floor value. (**b**) Normalized noise filter with IIR peak filter approximation defined by 6 dB bandwidth. (**c**) Magnetic noise density with minimum and IIR peak filter approximation.

The voltage noise amplitude spectral density (ASD) ${\hat{A}}_{\mathrm{vv}}\left(\Omega \right)$ (Figure 4a) is the other crucial performance metric obtained from the ME sensor characterization. It enables the estimation of the noise power at specific frequencies.

${\hat{A}}_{\mathrm{vv}}\left(\Omega \right)$ (Figure 4a) reaches its local maximum of 370 nV/$\sqrt{\mathrm{Hz}}$ at the sensor’s resonance frequency and approaches a floor level of approximately 50 nV/$\sqrt{\mathrm{Hz}}$. The filter characteristic is again obtained from the logarithmic noise density by normalizing it to the peak noise value in resonance:

(8) $${\hat{A}}_{\mathrm{vv}}^{\Omega_{0},\mathrm{dB}}\left(\Omega \right)=20\;\log_{10}\left(\frac{{\hat{A}}_{\mathrm{vv}}\left(\Omega \right)}{{\hat{A}}_{\mathrm{vv}}\left(\Omega_{0}\right)}\right).$$

The filter characteristic is approximated similarly to the frequency response by using an IIR peak (boost) filter [27] that employs a parallel allpass structure to limit the stopband’s attenuation. The required design parameters include the boost factor (ratio between passband and stopband) and the 6 dB bandwidth of 8.4 Hz (Figure 4b). The sensor’s performance in measuring magnetic fields (magnetic noise density ${\hat{A}}_{\mathrm{bb}}\left(\Omega \right)$) is dependent on both frequency response and voltage noise density:

(9) $${\hat{A}}_{\mathrm{bb}}\left(\Omega \right)=\frac{{\hat{A}}_{\mathrm{vv}}\left(\Omega \right)}{|\hat{H}\left(e^{j\Omega }\right)|}.$$

The resulting spectrum (c) presents a minimum in noise density of 4 pT at resonance with approximately linear slopes to both sides. Some form of magnetic noise density value (at a varying application-dependent frequency, e.g., [10]) is commonly used as an important performance metric for magnetometers. The curve is approximated by multiplying the inverse frequency response approximation and the voltage noise density approximation.

### 2.3. Signal Processing Structure

As previously characterized, the sensor offers a very limited 6 dB bandwidth of 12.5 Hz. Generally, it is beneficial for the SNR to adapt the bandwidth depending on the target application’s requirements. Thus, an equalizing filter corresponding to the inverse approximated frequency response was applied, which resulted in a flat frequency response in the region of interest. ${\hat{A}}_{\mathrm{bb}}\left(\Omega \right)$ (Figure 4c) is suitable for noise performance estimations, where a deviation from the sensor’s 6 dB bandwidth is required. This is practically realized by some form of band limiting.

The sensor also picked up a multitude of undesired magnetic and electric fields as it was operated unshielded in the experimental setup. This effect was even worsened by the forceful amplification of low frequencies due to the equalizer. It might be countered by a band limiting to the operating range of $f_{0}\pm 200$ Hz, which was subsequently applied using a 10th order Butterworth filter.

The final noise reduction step is a matched filter which corresponds to a bandwidth limitation and demodulation based on knowledge of the excitation signal g(n). The length of the matched filter $T_{\mathrm{mf}}$ (*N* samples) is an assumption on how long the desired signal d(n) is approximately constant. In the stationary case, one might choose a very long (e.g., multiple seconds) and therefore narrow matched filter to achieve a superior noise performance. In the 1D motion case (>0.1 m/s), the assumption of a constant d(n) for such a filter is invalid. Consequently, the matched filter distorts the estimation result $\hat{d}\left(k\right)$ by suppressing high frequency components of d(n).

**Figure 5:**
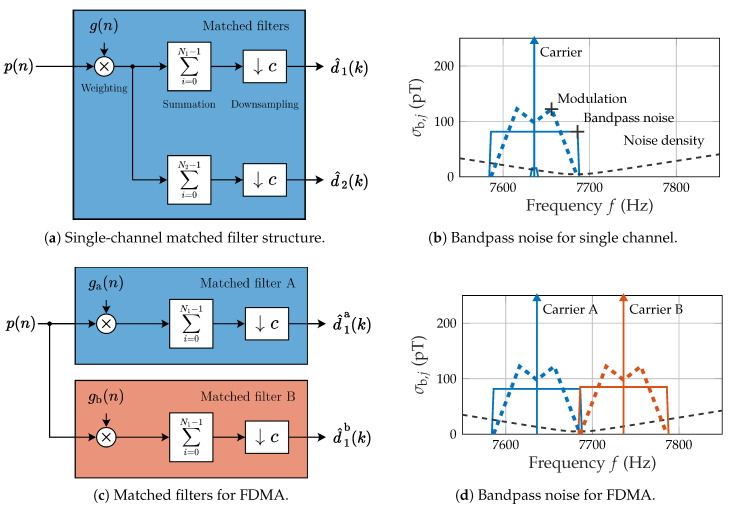
Matched filter structures and corresponding bandwidth considerations.

In general, the trade-off between a high spatial accuracy for slow movements (such as the localization of a resting object) and a high temporal accuracy for fast movements (such as obtaining the high-frequency components of movement) has to be managed. A matched filter implementation with the (equivalent) correlator realization [28] might be beneficial here, as it enables the parallel use of a multitude of filters with different lengths $N_{j}$. Thereby, the process of multiplying (weighting) the enhanced signal p(n) with the excitation signal g(n) can be separated from the summation and is only required once. The multiplication requires a matching phase (group delay for non-sinusoidal signals). Processing is done at a lower common sample (update) rate $f_{\mathrm{up}}$ (period: $T_{\mathrm{up}}$) of 100 Hz which is linked to the system sample rate $f_{s}$ of 48 kHz by the conversion factor *c*. Figure 5a illustrates this process in principle:

(10) $${\hat{d}}_{j}\left(k\right)=\frac{1}{N_{j}}\sum_{i=0}^{N_{j}-1}p\left(k-i\right)\;g\left(k-i\right)\;\mathrm{with}\;k=cn.$$

Our specific implementation for measurements (Figure 5a) covers two competing matched filters:

(1) $T_{\mathrm{mf},1}$ targets “fast” movements with a filter length of 10 ms, which corresponds to a two-sided bandwidth of 100 Hz.
(2) $T_{\mathrm{mf},2}$ targets “slow” movements with a filter length of 200 ms, which corresponds to a two-sided bandwidth of 5 Hz.

Integration of the magnetic noise density (ideal conditions, cf. Figure 4c) within the specified matched filter bandwidth illustrates the effect of the matched filter length (Figure 5b). The carrier is placed at 7636 Hz (approximately $f_{0}-$ 50 Hz). Modulation due to motion will require additional bandwidth apart from the carrier. Integration within an ideal bandpass is illustrated here as a rectangle with a width (integration bandwidth) and a height (square root of the integrated noise power $\sigma_{b,j}^{2}$). $T_{\mathrm{mf},1}$ yields an approximate theoretical noise of 80 pT, while $T_{\mathrm{mf},2}$ yields a significantly lower noise below 16 pT.

This matched filter implementation also allows for the separation of multiple simultaneously active carriers in a frequency division multiple access (FDMA) scheme (Figure 5c). The weighting signals $g_{a,b}\left(n\right)$ correspond to both active carriers. Subsequently, various matched filter lengths (e.g., $N_{1}$) might be applied in parallel. Figure 5d illustrates the placement of both carriers 100 Hz apart.

### 2.4. Simulation

In addition to the physical setup, we set up a simulation to provide a reference signal $\tilde{d}\left(k\right)$ for the estimation result $\hat{d}\left(k\right)$. As the processing strictly separates the excitation signal g(n) from the desired magnetic signal d(n), the simulation only includes a DC magnetic field based on a dipole approximation and an idealized 1D motion in this field:

(11) $$\tilde{d}\left(k\right)={\hat{\vec{e}}}_{s}\;\circ \;\underset{{\hat{b}}_{0}}{\underset{︸}{{\hat{a}}_{0}\;{\hat{I}}_{\mathrm{rms}}}}\;\;\cdot \;\;\frac{1}{{\hat{r}}^{3}}\;\;\cdot \;\;\vec{\Theta }\left({\hat{\vec{e}}}_{r}\right)\;\mathrm{with}\;\hat{\vec{r}}\left(k\right).$$

**Figure 6:**
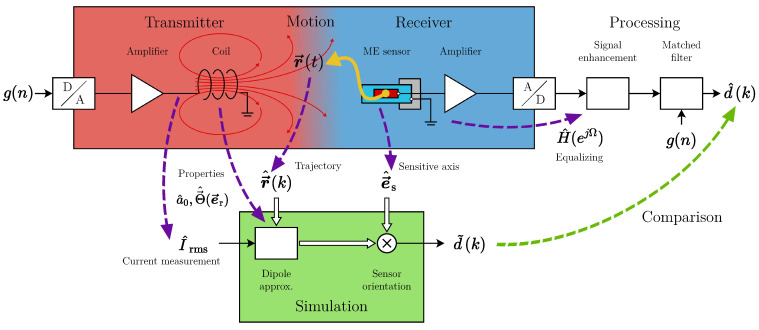
Overall signal chain including simulation.

Figure 6 visualizes how the previously characterized parameters of the overall system are included in the simulation model. In comparison to the physical setup, there is no bandpass filter applied, so even fast simulated movements will not lead to a signal distortion.

## 3. Measurements and Results

### 3.1. Measurement Setup

**Figure 7:**
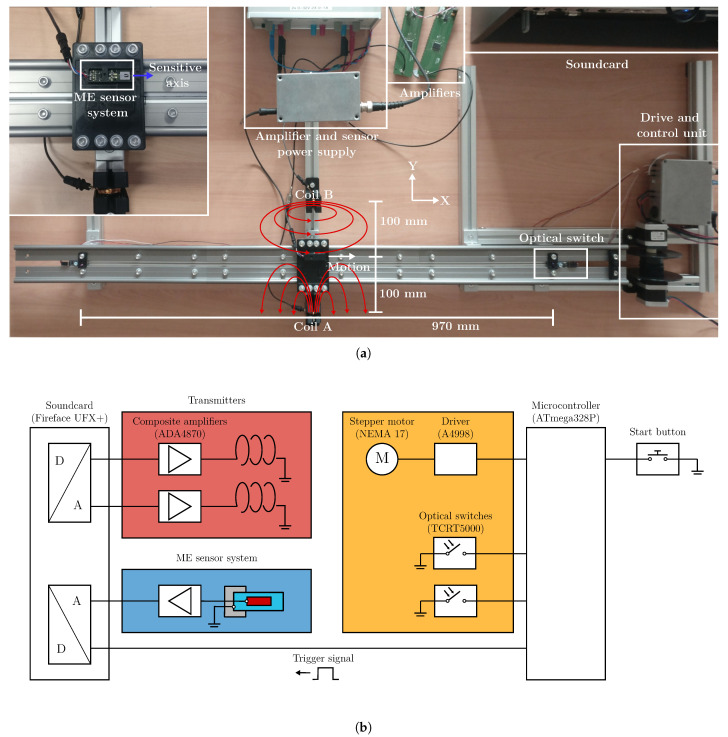
Experimental setup for magnetoelectric motion sensing. (**a**) Overview of the experimental setup. (**b**) Block diagram of key functional components.

Measurements were conducted in a simplified motion scenario (Figure 7) on a 1.2 m aluminum track. The sensor system is moving on a cart towed by stepper motors with its long axis ${\hat{\vec{e}}}_{s}$ in the direction of movement. The velocity $\dot{x}$ in the central part of the track (97 cm) is assumed to be constant. With a starting point $x_{0}$ and a $y_{0}$ displacement of 10 cm to each coil, the subsequent equation for the relative position is adopted for the field simulation:

(12) $$\hat{\vec{r}}\left(k\right)=\left(\begin{array}{c} x(k)\\ y_{0}\\ 0 \end{array}\right)=\left(\begin{array}{c} \dot{x}\;\cdot \;kT_{\mathrm{up}}+x_{0}\\ y_{0}\\ 0 \end{array}\right).$$

The setup features two cylindrical coils for magnetic excitation fields powered by two composite amplifiers (based on [29]) at a current ${\hat{I}}_{\mathrm{rms}}$ of approximately 400 mA. Optical switches close to the beginning and the end of the track enable synchronization and average velocity measurements. The conversion of signals between the analog and the digital domain was done using a sound card (UFX+, RME). The basic validity of the simulated magnetic flux density along the track was verified using a fluxgate sensor (FLC 100, Stefan Mayer Instruments).

### 3.2. Single Channel Measurements

**Figure 8:**
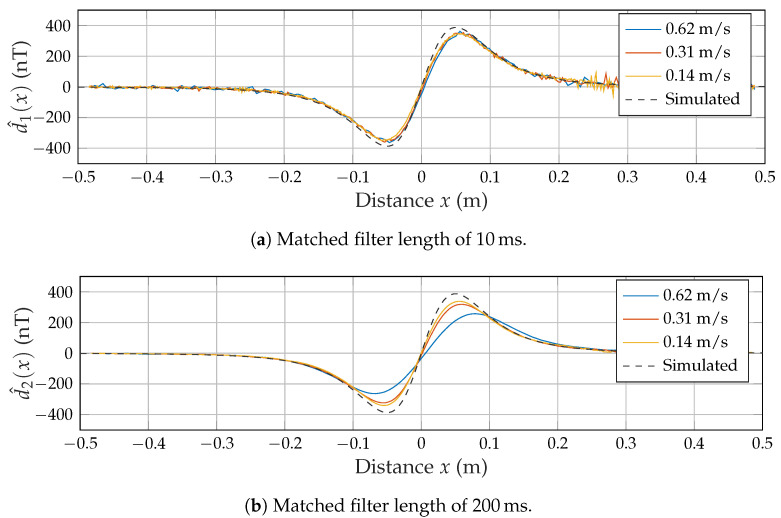
Measured signals for varying speeds and matched filter lengths.

As a first proof of concept, only coil A was used to generate a magnetic field, while the sensor was moving along the track multiple times at varying velocities. Based on the optical switches’ trigger signals, each desired signal’s time scale was rescaled to distance. As only the *x* position of the sensor is time-variant, we also refer to the measurement/simulation results as $\hat{d}\left(x\right)$ and $\tilde{d}\left(x\right)$, respectively. The previously introduced matched filter lengths $N_{1}$ and $N_{2}$ were applied on each signal to manage the trade-off between bandwidth and noise. Figure 8a shows accordance between the simulated signal and all three measured signals. However, the signals appear to be quite noisy. In Figure 8b, there is less noise but the signal measured at the fastest average velocity of 0.62 m/s is significantly flattened.

### 3.3. Noise Measurements

**Figure 9:**
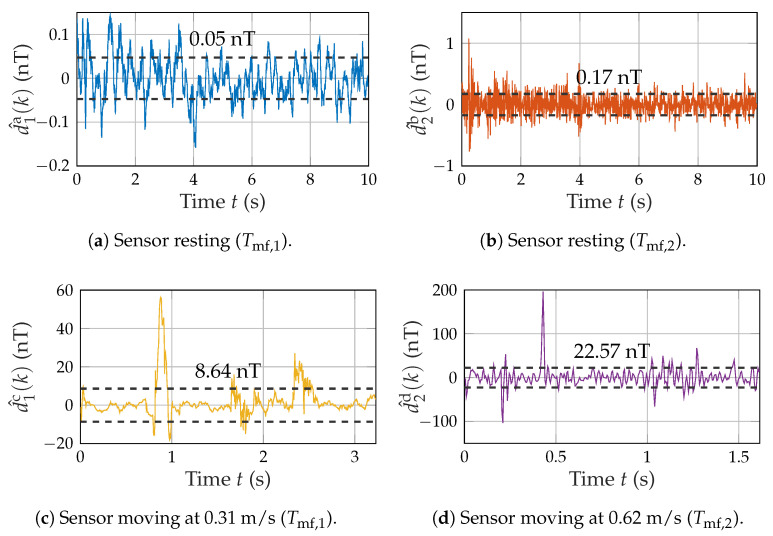
Noise measurements of moving and resting sensors. Each plot contains the noise at the matched filter output and the corresponding RMS value from $\sigma_{u,\;1}^{a}$ to $\sigma_{u,\;2}^{d}$.

As we established the receiver noise as the main limitation of system performance in the previous chapter, we conducted noise measurements (without magnetic excitation) of resting and moving sensors on the test setup (cf. Figure 8). Three different scenarios were tested and processed using the previously introduced matched filter of length $T_{\mathrm{mf},1}$ and $T_{\mathrm{mf},2}$, respectively. The resting case (Figure 9a,b) is thereby compared with a slower movement (Figure 9c) at the higher MF length and with a faster movement (Figure 9d) at the shorter MF length. For each measurement, the corresponding noise standard deviation (e.g., $\sigma_{u,\;1}^{a}$) is provided.

While the resting case ($\sigma_{u,\;1}^{a}$ and $\sigma_{u,\;2}^{b}$) shows a very low noise close to the spectral integration (cf. Figure 5b), the moving cases yield a significant increase in noise (approximately 100 times) due to spikes ($\sigma_{u,\;1}^{c}$ and $\sigma_{u,\;2}^{d}$). The assumption of a stationary noise power ($\sigma_{u}^{c},\;1$) during the motion is only valid for some sections of the time signal, as oscillation and multiple prominent peaks occur.

**Figure 10:**
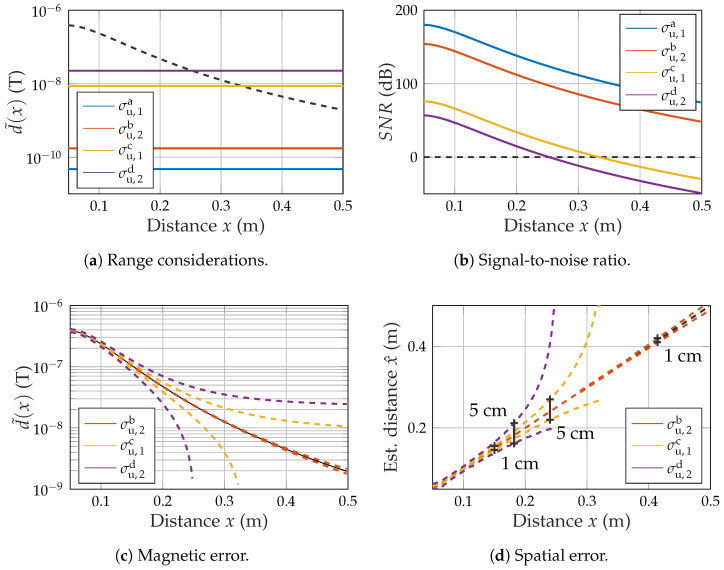
Performance metrics based on noise.

Based on the obtained noise values $\sigma_{u,\;1}^{a}$ to $\sigma_{u,\;2}^{d}$, multiple performance metrics regarding range and accuracy were calculated. Firstly, the noise floor was directly plotted in comparison to the positive half-wave of the simulated magnetic field (Figure 10a, cf. Figure 8). This leads directly to the SNR calculation as depicted in Figure 10b.

(13) $$SNR_{1}^{a}{\left(\vec{r}\right)}^{\mathrm{dB}}=20\log_{10}\left(\frac{\tilde{d}\left(\vec{r}\right)}{\sigma_{u,\;1}^{a}}\right)$$

While $\sigma_{u,\;1}^{a}$ and $\sigma_{u,\;2}^{b}$ show significant SNR levels even at at distance of 50 cm, the SNR reaches zero quickly for both other scenarios at 33 cm ($\sigma_{u,\;1}^{c}$) and 25 cm ($\sigma_{u,\;2}^{d}$), respectively. The noise can also be added on top of the simulated magnetic curve to obtain the uncertainty in the measurement of magnetic flux density for each position. This results in an upper and a lower boundary between which the measured value is to expected at the specified confidence of one standard deviation (Figure 10c). It is now possible to reverse search the corresponding spatial values $\hat{x}$ for the upper and the lower boundary at each position. This results in an upper and a lower estimated distance value. Error bars are included for relevant values of 1 and 5 cm (Figure 10d). While $\sigma_{u,\;2}^{b}$ ensures a high accuracy of 1 cm even at a distance of 42 cm, $\sigma_{u,\;1}^{c}$ reaches 1 cm at a distance of 15 cm and $\sigma_{u,\;2}^{d}$ never even reaches 1 cm.

### 3.4. Frequency Division Multiple Access Measurement

**Figure 11:**
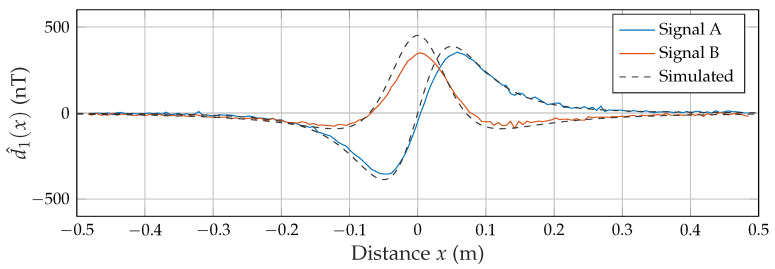
Estimated desired signals in an FDMA approach.

The FDMA approach utilizes the second excitation coil to transmit another carrier signal in a separate frequency band. As coil B is rotated by 90${}^{\circ }$ compared to coil A, the sensor measures the axial component of this magnetic field. Figure 11 shows the capability of the applied matched filter structure to smoothly separate both signals at the applied velocity of 0.31 m/s (with a matched filter length $T_{\mathrm{mf},1}$ of 5 ms) even with the high dynamic difference between both signals at a distance *x* close to zero.

## 4. Conclusions and Outlook

In this article, we presented a concept for magnetic motion sensing using ME sensors and multiple artificial sources. We focused on signal-to-noise ratio as the key metric to determine the performance of this approach and showed the basic feasibility in an experimental setup.

The setup was targeted at the demonstration of sensor measurements during a defined movement. While the restriction of motion to a single axis is suitable for characterization, it does not cover the full range of a 6D tracking problem, where position and orientation of the sensor might change. The solution of such a problem might require supplemental sensors and sources.

The first proof-of-concept measurements using a single or dual excitation signal generally show accordance with the magnetic simulation. A visual comparison between both scenarios shows no significant influence of intercarrier interference. While the magnetic simulation for coil A matched quite well, there was a more significant deviation for coil B. We generally expected some uncertainty for both coils regarding the exact measurement of the current and the estimation of position, orientation, and geometric properties of the coil (e.g., mean radius). In particular, the current might vary between each coil as well as between physical setup and simulation. This is due to the low impedance (cf. Figure 2), which is consequently affected by parasitic resistances of connectors and cables as well as measurement inaccuracy.

In contrast to the localization in the quasi-stationary case, we considered bandwidth as a key factor in motion sensing. The proposed structure uses multiple matched filters in parallel, which define the bandwidth requirements. The selection of the instantaneous bandwidth manages the trade-off between a high spatial accuracy for slow movements and a sufficient temporal accuracy for fast movements. There might be potential in an adaptive selection based on velocity or position estimations.

The noise measurements during motion showed a significant increase in noise compared to the resting case, which also leads to a degradation in SNR. Some of the noise-increasing effects might be related to the experimental nature of the measurement setup. As the sensor cable is trailing the cart, it might cause mechanical oscillations, which propagate to the sensor. This is relevant for the peaks that regularly occurred during the measurements. It also affected the theoretical range of 25 cm for the highest bandwidth, which was below our initial expectations. These performance metrics (cf. Figure 10) were obtained for an approximately constant movement. When considering the bandwidth only, they should be comparable for any arbitrary motion with similar bandwidth characteristics in its corresponding magnetic (desired) signal. However, there might be other mechanical effects in practical measurements that contribute to the additional noise.

As receiver noise was identified to be the key factor for system performance, there are multiple potential improvements conceivable: a cantilever without a magnetostrictive layer (piezoelectric sensor [30]) might be employed to analyze the effects of mechanical noise due to motion and apply adaptive noise reduction schemes; electrical shielding might improve noise performance at the cost of sensitivity and a tilted sensitivity axis (as in [9]); additionally, an increased matched filter length is an option for slow and quasi-stationary movements.

However, we assessed magnetoelectric motion sensing as a preliminary development stage of a comprehensive human motion tracking system. This does not yet allow for a meaningful comparison with common motion tracking systems. Instead, the SNR is more of a ground floor, which can potentially be significantly improved by subsequent tracking and sensor fusion algorithms (e.g., extended Kalman-based) if a priori knowledge on the motion and the moving object is taken into account.

All of the applied scenarios were focused on simple movements that are not generally comparable to the complexity of human movements. As we aim for an application in movement analysis for neurodegenerative diseases like Parkinson’s disease, an important next step is the application of a magnetoelectric motion sensing setup inside the clinical motor lab with our partners at the University Medical Center Schleswig-Holstein. State-of-the-art optical and IMU reference systems are available there to validate the magnetoelectric measurement results and evaluate the system’s performance. As the signal processing is currently done offline, such measurements would also greatly benefit from a real-time implementation.

## Data Availability

Not applicable.

## Abbreviations

The following abbreviations are used in this manuscript:

ME	Magnetoelectric
IMU	Inertial measurement unit
SNR	Signal-to-noise ratio
OPM	Optically pumped magnetometer
SQUID	Superconducting quantum interference device
DC	Direct current
AC	Alternating current
VLF	Very low frequency
TDMA	Time division multiple access
CDMA	Code division multiple access
FDMA	Frequency division multiple access
RMS	Root mean square
ASD	Amplitude spectral density
MF	Matched filter
